# Ectopic thyroid in the gallbladder accompanied with gallbladder adenoma: A case report

**DOI:** 10.1097/MD.0000000000018293

**Published:** 2019-12-16

**Authors:** Yanxu Li, Shijun Li, Meng Wang, Ling Tong

**Affiliations:** a Department of General Surgery, Chifeng Municipal Hospital, Chifeng Clinical Institute of Inner Mongolia Medical University, Chifeng 024000, Inner Mongolia Autonomous Region; b Department of Pathology, Chifeng Municipal Hospital, Chifeng Clinical Institute of Inner Mongolia Medical University, Chifeng 024000, Inner Mongolia Autonomous Region, China.

**Keywords:** adenoma, ectopic thyroid, gallbladder

## Abstract

**Rationale:**

Ectopic thyroid is most common in the tongue. Here we reported a rare case of thyroid tissue located in the gallbladder wall, accompanied with adenoma and a cyst lined with pseudostratified ciliated columnar epithelium in the neck region of gallbladder neck.

**Patient concerns:**

A 39-year-old female presented with recurrent upper abdominal pain and radiating back pain.

**Diagnoses:**

Based on ultrasonography, gallbladder polyps and calculous cholecystitis were suspected.

**Interventions:**

The patient was treated by laparoscopic cholecystectomy, and thyroid tissue located in the gallbladder wall was found. Histopathological examination showed no features of papillary thyroid neoplasm.

**Outcomes:**

The patient had no thyroid nodules or suspicious enlarged lymph nodes, and no other symptoms or complications by follow-up for 2.5 years up to September 2019.

**Lessons:**

We should pay attention to the rare location of ectopic thyroid tissue in the gallbladder and rule out primary thyroid malignancy to avoid unnecessary overtreatment.

## 1 Introduction

Ectopic thyroid is defined as non-neoplastic thyroid tissue found grossly or microscopically in a variety of locations aside from its normal site, usually for the abnormal descent of medial anlage of thyroid. Ectopic thyroid is most common in the tongue, in the females, especially in the Asian population, and it may occur in any age.^[1]^ A variety of unexpected locations of thyroid tissues have been reported including the gallbladder,^[2–9]^ lung,^[10]^ duodenum,^[11]^ porta hepatis,^[12]^ pancreas,^[13]^ adrenal gland,^[14]^ fallopian tube,^[15]^ and small intestinal mesentery.^[16]^ Here we reported a rare case of thyroid tissue located in the gallbladder wall, accompanied with adenoma and a cyst lined with pseudostratified ciliated columnar epithelium in the neck region of gallbladder neck. To our knowledge, this is the 9^th^ case of ectopic thyroid within or adjacent to the gallbladder.

## 2 Case report

A 39-year-old female presented with recurrent upper abdominal pain and radiating back pain was admitted to our hospital. She had these symptoms for 2 years, and the abdominal pain became progressive. The ultrasonography in local hospital 1 year ago showed a polyp with a diameter of 0.9 cm, which had grown to multiple polyps with the biggest one up to 1.7 × 1.8 cm, and there was muddy stones. Physical examination showed no significant signs. The preoperative diagnosis was gallbladder polyps and calculous cholecystitis. The patient underwent laparoscopic cholecystectomy. During the operation, we found that the gallbladder was small with slight edema, and there was a cystic nodule adhered to the neck of the gallbladder. Thyroid function test after the surgery showed normal results as follows: total T3 1.6 nmol/L (range 1.2–3.1 nmol/L), total T4 90.71 nmol/L (range 66–181 nmol/L), free T3 4.27 pmol/L (range 4.1–6.7 pmol/L), free T4 15.66 pmol/L (range 13.1–21.3 pmol/L), thyroid-stimulating hormone 2.4 mIU/L (range 0.27–4.2 mIU/L), thyroglobulin antibody < 10.0 IU/L (range 0–115 IU/L), thyroperoxidase antibody 9.52 IU/L (range 0–34 IU/L).

**Figure 1:**
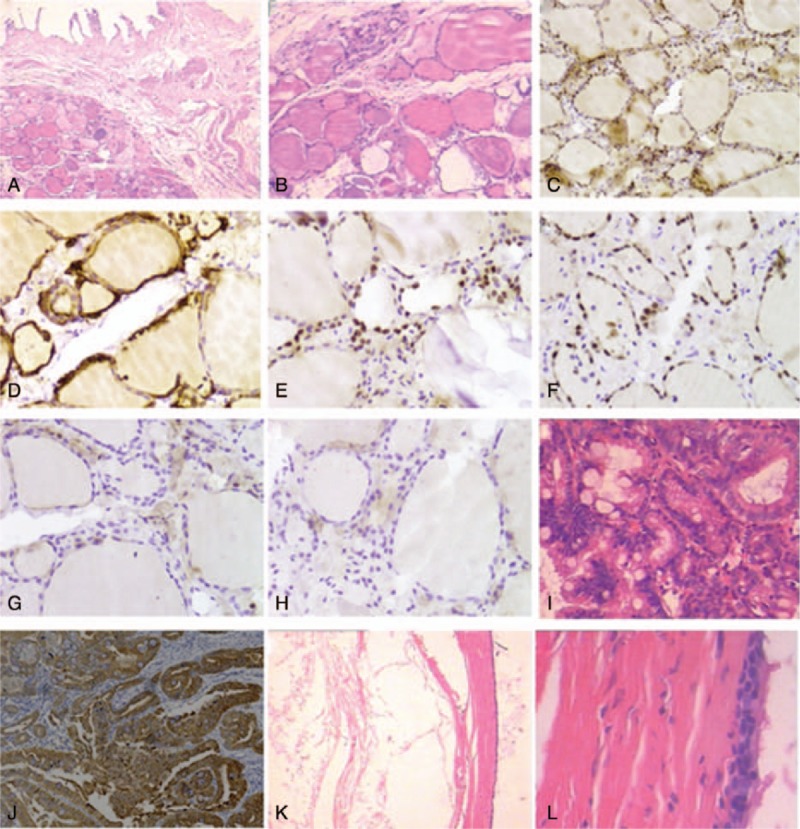
Ectopic thyroid tissue in the gallbladder wall. (A) The ectopic thyroid nodule was beneath the muscular layer of the gall bladder, and the epithelium of the gall bladder was atrophy. (B) The thyroid follicles lined by low cuboidal cells contained regular round nuclei and inconspious nucleoli, without the features of papillary thyroid neoplasm. (C–D) The ectopic thyroid showed cytoplasmic staining of thyroglobulin and TTF-1 in the follicular cell and the colloid. (E–F) Follicular cells immunostained for TTF-1 and PAX-8. (G–H) The follicular cells were negative for HBME-1 and CK19 staining. (I) The adenoma of the gallbladder. (J) The adenoma showed strong staining for CK19. (K) The cyst near cystic duct of gallbladder. Cyst lumen was present in left region. (L) Ciliated pseudostratified columnar epithelium of the cyst.

Gross examination showed that the gallbladder was 8.7 × 3.5 × 2.7 cm, the serosa of the gallbladder was smooth, and there was an anastomotic nail in the margin of the gallbladder. There were 5 polys up to 0.6 cm located in the body of the gallbladder. Gross examination did not reveal any abnormality suspicious for thyroid ectopia. The ectopic thyroid nodule was found in the perimuscular subserosal connective tissue in the body region of the gallbladder, and the epithelium of the gallbladder was atrophy (Fig. 1A). The thyroid follicles were lined by low cuboidal cells containing regular round nuclei and inconspious nucleoli. No features of papillary thyroid neoplasm were found (Fig. 1B). Immunohistochemistry staining for thyroglobulin and thyroid transcription factor-1 (TTF-1) of the ectopic thyroid tissue showed cytoplasmic staining of the follicular cell and the colloid (Fig. 1C, D). Follicular cells were positive for TTF-1 and PAX-8 staining (Fig. 1E, F), but were negative for hector battifora mesothelial antigen-1 (HBME-1) and cytokeratin-19 (CK-19) staining (Fig. 1G, H). Histopathology examination by hematoxylin and eosin staining revealed chronic inflammation in the gallbladder wall and multiple adenomas polyps in the mucosal epithelium (Fig. 1I). The adenoma showed strong CK19 staining (Fig. 1J).

There was moderate cell atypia of the largest polyp (Fig. 1K). The cyst near cystic duct of the gallbladder was lined with pseudostratified ciliated columnar epithelium (Fig. 1L). The patient had no thyroid nodules or suspicious enlarged lymph nodes, and no other symptoms or complications by follow-up for 2.5 years up to September 2019.

## 3 Discussion

Ectopic thyroid is usually found along the track from the floor of the primitive foregut to its final pretracheal position.^[5]^ Knowledge on embryologic development of the thyroid is the key to the understanding of the abnormalities of the thyroid gland, including the thyroid ectopia. In some locations, the presence of thyroid follicles can be explained as a metaplastic or heteroplastic phenomenon because the proximal segment of the gastrointestinal tract, liver, ventral pancreas, and the thyroid all share a common embryologic origin from the foregut endoderm.^[5]^

Ectopic thyroid in or adjacent to the gallbladder is extremely rare. We checked the literature and only 8 cases of ectopic thyroid tissue in or adjacent to the gallbladder have been reported up to now. Here we report a new case of ectopic tissue in the gallbladder wall, and review the literatures on ectopic thyroid tissue in the gallbladder as a clinical entity, to improve clinical diagnosis and management.

Including our case, there were only 5 cases of ectopic thyroid in the gallbladder wall: a case of thyroid tissue in the perimuscular subserosal connective tissue in the neck region of the gallbladder^[3]^; a case of the cystic mass filled with colloid like substance in the body and fundus region after excising subserosal layer^[6]^; a case of a small tan nodule in the neck of gallbladder around the cystic duct^[5]^; a case of thyroid tissue close to the cystic duct in the gallbladder wall and associated with gastric mucosal tissue^[9]^; and our case of thyroid tissue in the body of the gallbladder in the subserosal layer. For the other 4 cases: 1 case of thyroid tissue in fibrofatty tissue adjacent to the gallbladder^[12]^; 1 case of ectopic thyroid in the septum between the duplicated gallbladder^[7]^; 1 case of the nodule on the serosa aspect of the gallbladder^[8]^; and 1 case of an ectopic rest adjacent to the gallbladder.^[4]^ The embryological defect that may cause ectopic thyroid in the gallbladder remains to be investigated. A dual differentiation from a common potential endodermal cell precursor of the gut may be the best explanation.^[2]^

In the thyroid ectopia of gallbladder, women are more frequently affected than men (7 versus 2), and the age of the patients ranged from 29 to 76 years (mean age 52.5). The size of ectopic thyroid in the gallbladder ranged from 2 to 30 mm (mean size 12.4 mm), sometimes with a cystic-solid mass. The symptoms of ectopic thyroid in the gallbladder are usually presented as recurrent right upper quadrant pain resulting from chronic or acute cholecystitis. They are usually detected incidentally by histopathology examination after the cholecystectomy. Only 2 cases were found before the surgery. The prevalence of ectopic thyroid in the gallbladder is not estimated. Preoperative clinical examinations are not valuable for the diagnosis of ectopic thyroid in the gallbladder.

In general, differential diagnosis of thyroid tissue in abnormal locations includes metastatic carcinoma, nonneoplastic thyroid tissue, thyroid neoplasia arising in ectopic tissue, and teratoma. It is important to determine whether the thyroid tissue found in or adjacent to the gallbladder is ectopic or a metastasis from a papillary or follicular carcinoma of the thyroid.^[2]^ Although distinguishing between the metastatic and truly ectopic thyroid tissue can be challenging, we can examine malignant thyroid neoplasia history, the abnormalities of thyroid or neck nodules, and histopathology findings of the papillary thyroid carcinoma. In addition, immunohistochemistry staining and molecular detection may help achieve the definitive diagnosis.^[17]^ Thyroglobulin, TTF-1, thyroid peroxidase, cytokeratin-19, HBME-1, and PAX-8 may exhibit different expression on the ectopic tissue and thyroid neoplasia. The application of molecular analysis to detect V600E (1799T>A) BRAF gene mutation and KRAS gene (codons 12, 13, 61) mutation is also helpful. Currently, there is no consensus on optimal therapeutic strategy for ectopic thyroid, perhaps due to the rarity of this clinical entity.

In conclusion, we present a rare case of ectopic thyroid in the gallbladder wall. It is important to pay attention to the rare location of ectopic thyroid tissue and rule out primary thyroid malignancy to avoid unnecessary overtreatment.

## Author contributions

**Investigation:** ling tong, Yanxu Li, Shijun Li, Meng Wang.

**Writing – original draft:** ling tong.
